# Analysis of the Skew Deviation to Evaluate the Period of Onset of a Canalolithiasis After Macular Damage

**DOI:** 10.3389/fneur.2020.572531

**Published:** 2020-10-23

**Authors:** Mauro Gufoni, Matteo Vianini, Augusto Pietro Casani

**Affiliations:** Department of Surgical Pathology, Medical, Molecular and Critical Area, Otolaryngology Section, University of Pisa, Pisa, Italy

**Keywords:** vertigo, benign paroxismal positional vertigo, ocular tilt reaction, skew deviation, vestibular disorder (VD)

## Abstract

Benign paroxysmal positional vertigo (BPPV) is the most common peripheral vestibular end-organ disease, and it is one of the first causes of access to the emergency room. The moment of migration of the otoconial debris in a semicircular canal does not necessarily coincide with the moment of detachment of the debris themselves. Consequently, the paroxysmal positional vertigo could arise with a variable delay with respect to the mechanical damage suffered by the macula. The aim of this work is to try to identify objective criteria to establish whether a canalolithiasis is synchronous or diachronic to the damage. The analysis of skew deviation in the context of ocular tilt reaction in patients with canalolithiasis could provide useful information to understand if macular damage occurred at the origin of the disease and when the damage may have occurred. In this study, 38 patients with BPPV were analyzed based on the type of skew deviation that was presented. We found that if the eye on the side of the canalolithiasis is hypotropic the damage of the utriculus is likely recent (last 10 days), if it is hypertropic the damage is not recent (20 days before) and finally if the eyes are at the same height it could be an utricular damage in compensation (occurring the last 10–20 days) or a secondary labyrinth canalolithiasis, without associated utricular damage. Our results show that the evaluation of skew deviation in patients suffering from BPPV could be useful to evaluate: (a) if a positional paroxysmal nystagmus can be related to an previous relevant injury event (for example a head injury that occurred days before the crisis); (b) if it is a BPPV of recent onset or a re-entry of the debris into the canal.

## Introduction

Benign paroxysmal positional vertigo (BPPV) is the most common peripheral vestibular end-organ disease and it is characterized by a sudden, transient vertigo which is accompanied by the typical paroxysmal positional nystagmus. Symptoms are provoked by positional changes of the head with respect to gravity and can range in severity from mild dizziness to debilitating episodes that may induce nausea or vomiting, and significantly reduced quality of life (1). BPPV is caused by otoconia that have become detached from the utricular maculae. The otoconia could enter the semicircular canal and can move in the endolymph (canalolithiasis) or become attached to the cupula (cupulolithiasis). Nowadays, the availability of neurophysiological tests such as cervical and ocular Vestibular Myogenic Potentials, provide the possibility to detect the functionality of the utricular and saccular maculae. By the above-mentioned tests, a damage of the utricular and saccular macula could be detected in BPPV (2, 3). Skew deviation (SD) is a vertical misalignment of the eyes caused by damage to prenuclear vestibular input to ocular motor nuclei. It is usually accompanied by binocular torsion, torticollis, and a tilt in the subjective visual vertical. This constellation of findings has been termed as ocular tilt reaction (OTR). SD can result from any acute injury within the posterior fossa, the majority of cases are seen in association with brainstem stroke. A tonic ipsiversive ocular tilt reaction, not associated to a central nervous system damage, could be related to a damage to one utricle or other lesions involving the human labyrinth and vestibular nerve (4, 5). An injury of one utricular macula could produce an otolithic detachment but the moment of migration of the otoconial debris in a semicircular canal does not necessarily match with the moment of detachment of the debris themselves. We hypothesize that the study of the skew deviation could give important information regarding the timing of the onset of a paroxysmal positional vertigo. The aim of this paper is to identify objective criteria to establish whether a BPPV occurs at the same time as the damage of utricle or subsequently.

## Methods

**Figure 1:**
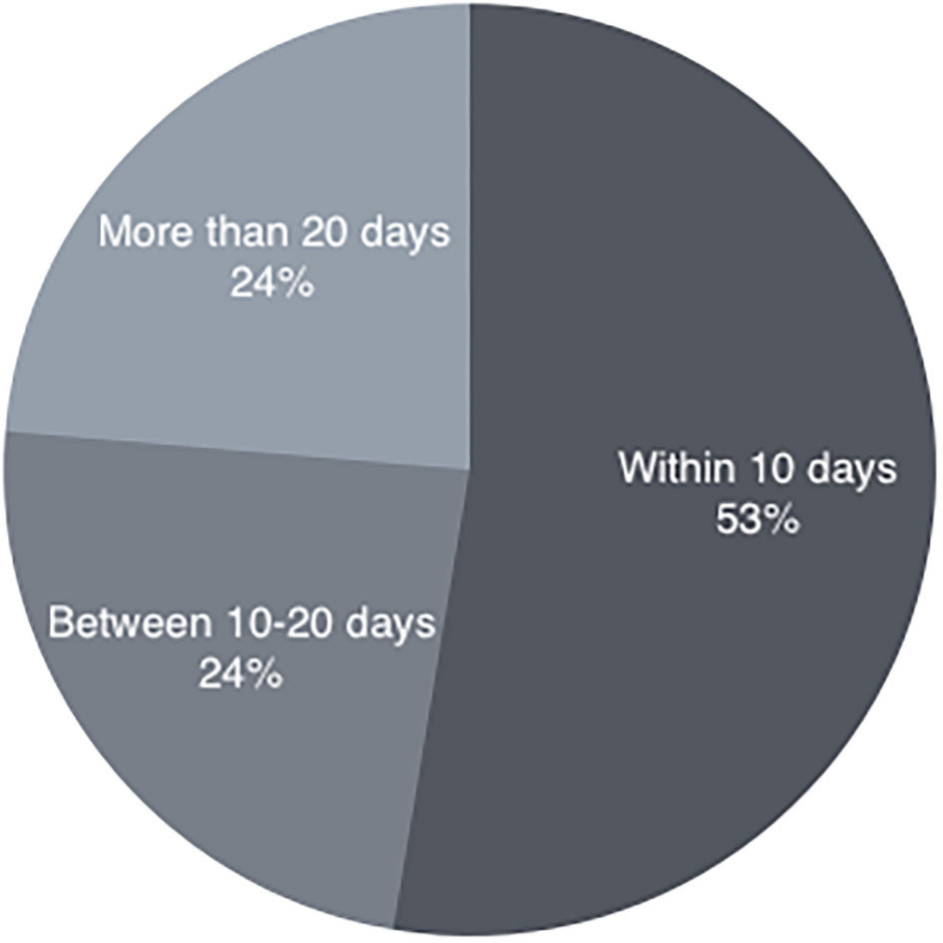
Time of onset of BPPV.

**Figure 2:**
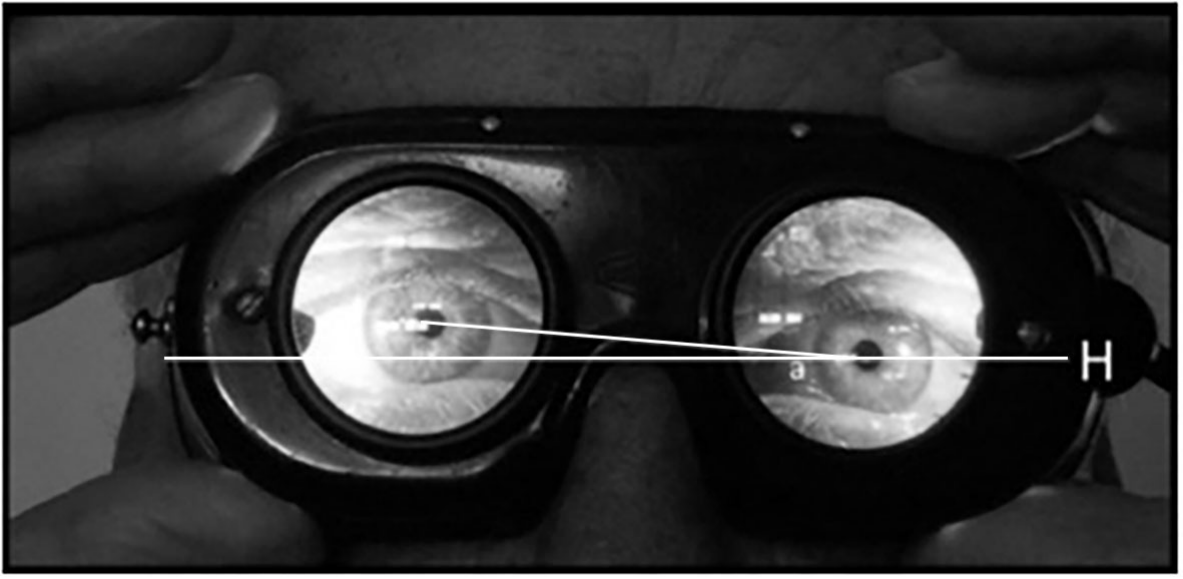
Evaluation of the angle “a” between interpupillary line and horizon: in this case the angle is 5°.

In the period from January 2018 to January 2020 we consecutively evaluated 120 patients with BPPV. Our inclusion criteria were based on the presence of recurrent short-lasting episodes of positional vertigo, on the positivity of the provocation maneuvers, and on nystagmus characteristics (latency, fatigue and direction) compatible with BPPV. The diagnosis of posterior canal BPPV was made using the Dix–Hallpike test that elicited a mixed vertical– torsional Paroxysmal positional nystagmus with the vertical component beating toward the forehead, and the upper pole of the eyes beating toward the affected (lower) ear. We used the Pagnini-McClure maneuver to diagnose horizontal semicircular canal BPPV. This was considered positive when a horizontal direction-changing nystagmus (geotropic or apogeotropic) appeared; the directions of lying-down nystagmus and head-bending nystagmus have been used to correctly evaluate the affected side (6). All the patients were carefully asked to clearly verify the exact day of onset of the symptomatology. They were successfully treated for complete resolution of nystagmus after appropriate repositioning maneuver; anamnesis of the exact day of the onset of the BPPV symptoms. We included in our study 38 subjects (16 males and 22 females, mean age 58 years, ranging from 15 to 90 years) successfully treated with the repositioning maneuver. Twenty-seven subjects, suffering from posterior canal BPPV were treated with the Semont's maneuver and 11 subjects, suffering from lateral canal BPPV were treated with the Gufoni's maneuver. We excluded patients who presented with multiple canal BPPV, with a prevalent downbeating nystagmus, or patients with no immediate response to the therapeutic maneuver. We excluded patients with associated vestibular pathology (such as previous acute unilateral loss, Menière's Disease) or signs of central nervous system involvement and patients affected with ophthalmological diseases. The selected patients were divided into three groups (Figure 1): (a) canalolithiasis recently occurred: within 10 days before clinical observation (b) canalolithiasis not recently occurred: between 10 and 20 days before clinical observation (c) canalolithiasis that occurred late: more than 20 days before clinical observation. The evaluation of the skew deviation has been performed with Frenzel Goggles and its measurement with the Eye Alignment Test. The test includes these steps: (1) The patient is seated and wears Frenzel's glasses, without resting his back or his arms. (2) The examiner takes a photo of the eyes: the camera is equipped with “a level” to keep it perfectly horizontal. (3) The angle between the interpupillary line and the horizontal line is measured on the photo (Figure 2). Some “vector graphics software” can be used. The inclination of the interpupillary line is measured and it is considered pathological if the result is an angle >2.5° (7). The side from which the eye is lower is identified and the records relating to each patient were noted. The examiner who carried out the measurement of the eyes was different from the examiner who had carried out the diagnosis of canalolithiasis and did not know which was the pathological side. All patients underwent routinely-performed tests only, without invasive or experimental procedures. Informed consent was obtained from all participants and the study was performed in accordance with the Declaration of Helsinki.

## Results

**Figure 3:**
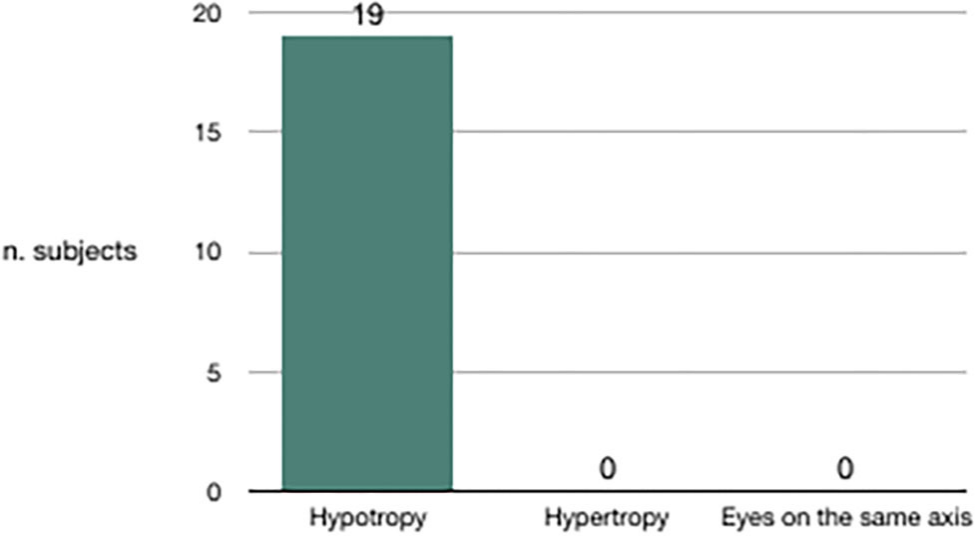
Subjects with BPPV occurred within 10 days before clinical observation.

**Figure 4:**
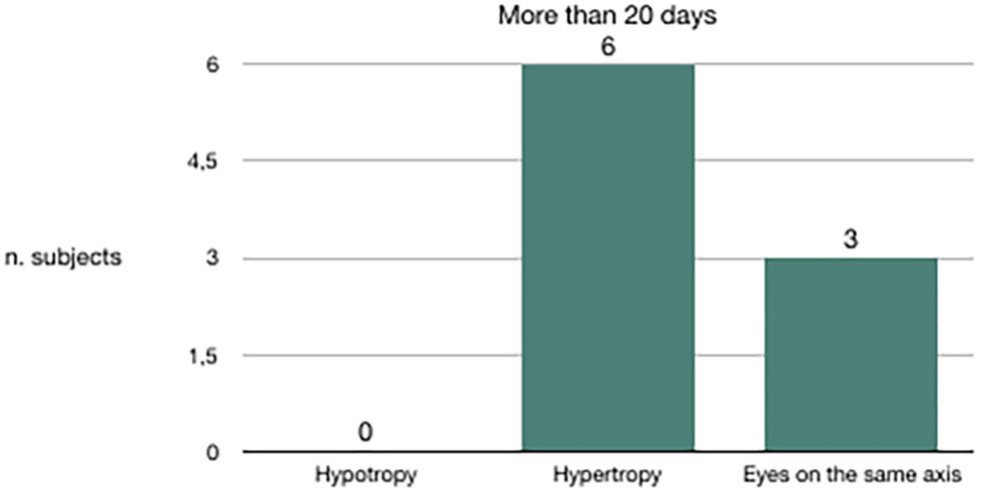
Subjects with BPPV occurred more than 20 days before clinical observation.

**Figure 5:**
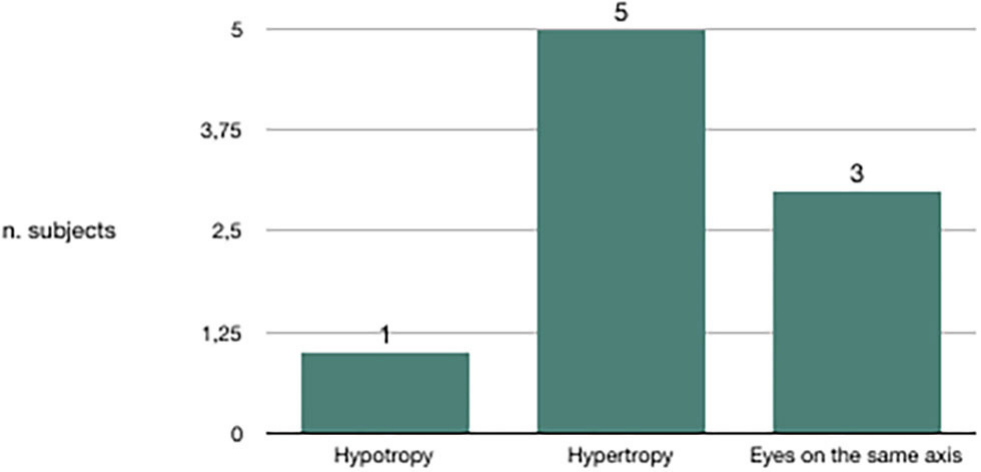
Subjects with BPPV occurred 10–20 days before clinical observation.

We evaluated the congruence between the BPPV affected side and the skew deviation. Out of 20 subjects in whom canalolithiasis had occurred 10 days before clinical observation, 19 had hypotropia of the eye on the same side of the affected vestibule (Figure 3). Out of nine subjects in whom canalolithiasis had occurred 20 days before clinical observation, six had hypertropia of the eye on the same side of the affected vestibule (Figure 4). Out of 9 subjects in whom canalolithiasis had occurred between 10 and 20 days before clinical observation: three had compensated skew deviation (eyes on the same axis), one had uncompensated skew deviation (hypotropia of the eye on the same side of the affected vestibule), five had hyper compensated skew deviation (hypertropia of the eye from the sick side) (Figure 5). A comparison was made (Chi-squared test) for the presence of hypertropia of the eye from the side of the damaged labyrinth between two groups: a group with recent onset of canalolithiasis (<10 days) and a group with canalolithiasis present for at least 20 days. The difference was statistically significant (*p* = 0.00033) allowing the zero hypothesis of belonging to the same group to be rejected.

## Discussion

From the evidence of this study, it therefore seems possible to trace the age of onset of macular damage on the basis of the degree of compensation of the ocular tilt reaction, with particular reference to skew deviation. If the eye on the side of the canalolithiasis is hypotropic the damage of the utriculus is likely recent: last 10 days. If the eye on the side of the canalolithiasis is hypertropic, the damage of the utriculus is not recent: dates back to at least 20 days before. If the eyes are at the same height it could be: (a) a secondary labyrinth canalolithiasis, without associated utricular damage. (b) utricular damage in compensation: occurring in the last 10–20 days. In literature, OTR is assessed using different methods, which have as their purpose the measurement of at least one of the three signs that compose it (ex/inciclotorsion, hyper/hypotropia of the eye, lateral flexion of the head). One method is the evaluation of the subjective visual vertical (SVV) (8): the angle at which a light line in the dark is perceived to be perfectly vertical is measured. A dynamic SVV toward the affected side was recorded in all subtypes of BPPV with a statistically significant difference from those of the controls (9). A variant that does not require instrumentation is the “Bucket test” (7). Using this method, a reduction of the deviation of SVV was detected immediately after the repositioning maneuvers (10). A complete study of OTR should be based on the evaluation of the three signs of OTR at the same time, including the exiclo and inciclo torsion of the eyes. This component of OTR is not uniquely considered an expression of macular damage (11–15) and could generate a “bias” in the evaluation of the utricular function. The ocular Vemps (oVemps) are generated from the utricle and carried along the otolith-ocular crossed neural pathways (3). A recent meta-analysis indicated that in patients with BPPV, oVemps showed an abnormal asymmetry ratio reflecting a difference between affected and not affected sides (2). However, the same authors suggested that this method is not suitable for clinical application.

Our study has several limitations: firstly, the sample size is too small to drive a definitive conclusion; secondly, comparing the results of OTR in our patients with the results of o-Vemps would have provided more detailed information about the presence of a utricular dysfunction. Nevertheless, the aim of our study was not the evaluation of the functionality of the utricular macula, but to obtain reliable data about the restoration of the macular damage.

## Conclusions

Analyzing skew deviation in the context of ocular tilt reaction in patients who come to our clinical attention with canalolithiasis can provide extremely useful information to help understand if macular damage occurred at the origin of the disease and when the damage may have occurred. We are of the opinion that this method can be useful to evaluate if a positional paroxysmal nystagmus can be related to an anamnestically relevant injury event or if it is a new BPPV or a re-entry in the channel of pre-existing otoliths.

## Data Availability Statement

The raw data supporting the conclusions of this article will be made available by the authors, without undue reservation.

## Ethics Statement

Ethical review and approval was not required for the study on human participants in accordance with the local legislation and institutional requirements. The patients/participants provided their written informed consent to participate in this study.

## Author Contributions

MG: study concept, design, acquisition of data, analysis, interpretation of data, drafting of the manuscript, and study supervision. MV: acquisition of data, drafting of the manuscript, administrative support, and technical and material support. AC: study concept, design, analysis, interpretation of data, drafting of the manuscript, and study supervision. All authors contributed to the article and approved the submitted version.

## Conflict of Interest

The authors declare that the research was conducted in the absence of any commercial or financial relationships that could be construed as a potential conflict of interest.
